# Predictors of renal and patient survival in lupus nephritis: a 28-year single-center cohort study

**DOI:** 10.3389/fneph.2026.1945616

**Published:** 2026-09-25

**Authors:** Alexandra Vrabie, Bogdan Obrişcă, Bogdan Sorohan, Roxana Jurubiţă, Camelia Achim, Diana Silvia Zilişteanu, Andreea Gabriela Andronesi, Gabriela Lupuşoru, Elena Emanuela Rusu, Raluca Bobeică, Elena Georgia Micu, Sonia Bălănică, Andreea Berechet, Valentin Mocanu, Ştefan Lujinschi, Nicu Caceaune, Gener Ismail

**Affiliations:** 1 Nephrology Department, “Carol Davila” University of Medicine and Pharmacy, Bucharest, Romania; 2 Nephrology Department, Braşov Emergency Clinical Hospital, Braşov, Romania; 3 Nephrology Department, Fundeni Clinical Institute, Bucharest, Romania; 4 Kidney Transplantation Department, Fundeni Clinical Institute, Bucharest, Romania; 5 Internal Medicine Department, Fundeni Clinical Institute, Bucharest, Romania

**Keywords:** chronic kidney disease, end stage kidney disease, lupus nephritis, prognostic score, systemic lupus erythematosus

## Abstract

**Introduction:**

Despite advances in the understanding and treatment of lupus nephritis (LN), long-term renal and patient outcomes remain suboptimal. Identifying reliable predictors of prognosis and early treatment response is essential for improving risk stratification and guiding management.

**Methods:**

We conducted a retrospective cohort study of patients with biopsy-proven LN. Long-term renal and patient survival, renal response at 6 and 12 months, and predictors of a composite outcome (doubling of serum creatinine, end-stage kidney disease [ESKD], or death) were evaluated.

**Results:**

A total of 147 patients were included, with a median follow-up of 39.5 months. 10.3% of patients had a doubling of serum creatinine, 11.0% progressed to ESKD, and 6.8% died. Proteinuria <0.7 g/day was achieved by 49.1% and 54.4% of patients at 6 and 12 months, respectively. In multivariate analysis, baseline predictors of the composite outcome included age >50 years, proteinuria ≥3 g/day, and hemoglobin <10 g/dL, while an activity index ≥9 and low baseline C4 levels showed a protective trend. Achieving proteinuria <0.7 g/day at 12 months emerged as the strongest protective factor. A prognostic score demonstrated good discrimination (area under the curve [AUC] 0.82), which improved further with the addition of proteinuria at 12 months (AUC 0.86).

**Conclusions:**

In this biopsy-proven LN cohort, early renal response, particularly proteinuria <0.7 g/day at 12 months, was the strongest predictor of long-term renal and patient survival. These findings support a dynamic, response-based approach to prognostication in LN and provide the basis for a clinically applicable prognostic score that requires external validation.

## 1 Introduction

Systemic lupus erythematosus (SLE) is an autoimmune disease with frequent kidney involvement, lupus nephritis (LN) occurring in up to 60% of patients (1, 2). Despite major advances in the understanding of LN pathophysiology and the introduction of newer therapeutic strategies, morbidity and mortality among LN patients remain substantial. Preserving renal function, preventing progression to end-stage kidney disease (ESKD), and reducing mortality continue to represent the primary goals of LN management (2, 3).

Recent clinical trials have reported complete renal response (CRR) rates at 52–76 weeks, ranging between 35% and 46%, while primary efficacy renal response (PERR) was achieved in approximately half of patients in pivotal studies such as BLISS-LN (4, 5). Importantly, achieving and maintaining a sustained renal response has been consistently associated with improved long-term renal and patient survival (4–7).

Consistent with the current suboptimal renal response rates, in the developed countries, up to 33% of the proliferative class IV LN patients still progress to ESKD within 10 years from the diagnosis based on a meta-analysis with studies from the late 2000s (8). Conversely, a multicentric Italian LN cohort published in 2018 that included 76.8% proliferative LN patients reported an increase in the ESKD-free survival at 10 years from 87% in the period of 1970–1985 to 99% in the period of 2002-2016 (3). Preventing the development of ESKD is important in SLE patients, as reaching ESKD is a risk factor for death in these patients, leading to a 26-fold increase in the mortality risk (9). Fortunately, the 5-year general survival in LN patients has improved constantly, from 44% in the 1950s to nearly 94% in the present day (10, 11).

The identification of reliable predictors of long-term renal and patient outcomes represents an unmet need in LN management. Traditional clinical, laboratory, and histopathological markers—including proteinuria, renal function, complement levels, and activity and chronicity indices—have demonstrated variable prognostic value across cohorts (3, 5, 12–16). Although several histologic and serologic biomarkers have been proposed, no single marker has consistently shown sufficient accuracy to guide individualized risk stratification (12, 13). As a result, recent efforts have focused on the development of multivariable prediction models, which require validation in independent cohorts before clinical implementation (17, 18).

In this study, we aimed to characterize a large cohort of patients with biopsy-proven LN, evaluate modality of presentation, treatment patterns, renal response rates at 6 and 12 months, and identify predictors of long-term renal and patient survival.

## 2 Materials and methods

### 2.1 Study design and population

We performed a retrospective, observational study on all patients with SLE and first biopsy-proven episode of LN followed in the Nephrology Department of Fundeni Clinical Institute from Bucharest, Romania, between 1997 and 2025. The diagnosis of SLE was made according to the 2012 Systemic Lupus International Collaborating Clinics (SLICC) (19) or 2019 European League Against Rheumatism (EULAR)/American College of Rheumatology (ACR) criteria (20). The inclusion criteria were histologic confirmation of LN and biopsy fragment adequate for assessment of histologic activity and chronicity. We excluded patients with other glomerular disorders and those with insufficient clinical data. Kidney biopsies were classified according to the International Society of Nephrology (ISN)/Renal Pathology Society (RPS) LN criteria and scored according to the modified National Institutes of Health (NIH) activity and chronicity indices (21).

The clinical and laboratory parameters were obtained by reviewing the patient’s electronic medical records at the time of kidney biopsy, at 6 months, at 12 months and at the follow-up and included: interval between the diagnosis of SLE and the diagnosis of LN, age, gender, renal function (serum creatinine and estimated glomerular filtration rate (eGFR) according to the 2009 CKD-EPI equation), serum albumin (g/dl), hemoglobin (Hb) (g/dl), proteinuria/24-hours (g/day), hematuria (cells/µL), C3 and C4 levels (mg/dL) and the presence of antiphospholipid syndrome. LN patients received immunosuppressive therapy in accordance with the EULAR or KDIGO treatment guidelines (22, 23). Data on death were obtained from electronic medical records for patients who died in the hospital and through information obtained from relatives for patients who died outside the hospital.

This study was conducted with the provision of the Declaration of Helsinki, and the protocol was approved by the local ethics committee (The Ethics Council of Fundeni Clinical Institute; Registration number: 53068/12.12.2025). The need for informed consent was waived due to the exclusive use of deidentified information and the retrospective nature of the study.

### 2.2 Study outcomes

The primary outcome was a composite of doubling of serum creatinine, ESKD, or death from any cause, whichever occurred first. The secondary outcomes were: doubling of serum creatinine (defined as persistent doubling of the lowest serum creatinine level attained on follow-up), ESKD (defined as the need for chronic renal replacement therapy, either dialysis or transplant), death from any cause, and the achievement of proteinuria of 0.7 g/day or less at 6 and 12 months.

The *post hoc* analysis of the Euro-Lupus Nephritis and MAINTAIN Nephritis trials supports the use of proteinuria criterion of the primary efficacy renal response (PERR), defined as a urinary protein-creatinine ratio of ≤0.7 g/g (24). In our retrospective database, proteinuria values were primarily recorded as 24-hour proteinuria (g/24h). To harmonize the data, 24-hour proteinuria values were considered equivalent to urinary protein-creatinine ratio, based on the assumption that the average creatinine excretion rate is approximately 1.0g/24h (25). No formal mathematical conversion formula was applied between the two measures.

### 2.3 Statistical analysis

Continuous variables were expressed as either mean (± standard deviation (SD)) or median (interquartile range (IQR): 25th–75th percentiles), according to the distribution of the variables, and categorical variables as percentages. Differences between groups were assessed in the case of continuous variables by the Mann–Whitney test. Categorical variables were compared between groups using the chi-square test. The probability of ESKD-free survival and patient survival was assessed by the Kaplan-Meier method, and the log-rank test was used for comparisons. Univariate and multivariate Cox proportional hazard regression analyses were performed to identify independent predictors of the composite outcome. The results of Cox analyses are expressed as a hazard ratio (HR) and 95% confidence interval (95% CI). In multivariate analysis, a backward stepwise elimination algorithm was applied. All variables from the univariate analysis were entered into the initial model. At each step, variables were progressively removed from the model based on the highest p-value. Patients with missing values for variables included in the regression analysis were excluded from the corresponding multivariable analysis. The performance of the prediction model was assessed by the receiver operating characteristic curve (area under the curve (AUC)). In all analyses, p values are two-tailed, and all p values less than 0.05 were considered statistically significant. Statistical analyses were performed using the SPSS program (SPSS version 26, Chicago, IL, USA) and GraphPad Prism version 9.3.1 (1992–2021 GraphPad Software, LLC).

## 3 Results

### 3.1 Patient baseline characteristics

Of the 155 patients assessed for eligibility, 147 were included in the final analysis after excluding 8 patients (3 with other glomerular disorders and 5 with insufficient clinical data). The study cohort included 147 patients with a median age of 33 years (IQR:24-43), 80.3% being females. The median interval between the diagnosis of SLE and the diagnosis of LN was 2 years. The distribution of the indications for biopsy of the patients was the following: 37.33% with chronic nephritic syndrome, 27.47% with nephrotic syndrome with active urinary sediment, 21.12% with isolated proteinuria, 8.45% with nephrotic syndrome, 4.22% with isolated hematuria, and 1.41% with acute nephritic syndrome.

The median eGFR was 77 ml/min/1.73 m^2^ (IQR:55.5-108), 31.3% of the patients having an eGFR of less than 60 ml/min/1.73 m^2^. The median proteinuria was 1.98 g/day (IQR: 0.8-4.76), 37.4% of the patients having a proteinuria of more than 3g/day. 24.5% of the cohort had secondary antiphospholipid antibody syndrome. In terms of complement levels, 59% and 28.4% of the study cohort had a low baseline C3 and C4, respectively. All the baseline characteristics are summarized in **Table 1**.

**Table 1:** Baseline characteristics of the study cohort.

Variable	Value
Number of patients	147
Age (years)	33 (IQR: 24-43)
Age over 50 years (%)	17.7%
Gender (%F/%M)	80.3%/19.7%
Time since SLE onset (years)	2 (IQR:0.3-8.75)
Antiphospholipid syndrome (%)	24.5%
Serum creatinine (mg/dL)	1 (IQR:0.8-1.32)
eGFR (mL/min/1.73 m^2^)	77 (IQR:55.5-108)
• eGFR < 30 mL/min (%).	8.8%
• eGFR < 60 mL/min (%).	31.3%
Serum albumin (g/dL)	3.7 (IQR:3.2-4.1)
Serum albumin < 3 g/dL (%)	17.1%
Hemoglobin (g/dL)	11.9 (IQR:9.8-12.8)
Hemoglobin < 10 g/dL (%)	28.1%
24-h proteinuria (g/day)	1.98 (IQR:0.8-4.76)
24-h proteinuria (%)	
• < 1 g/day.	28.1%
• 1–3 g/day.	34.5%
• >3 g/day.	37.4%
Microscopic hematuria (%)	66.4%
Serum C3 (mg/dL)	83 (IQR:64-102)
Low serum C3 (%)	59%
Serum C4 (mg/dL)	15.3 (IQR:8-22)
Low serum C4 (%)	28.4%
Follow-up (months)	39.5 (IQR:16.7-72)

dL, deciliter; eGFR, estimated glomerular filtration rate; F, female; g, gram; IQR, interquartile range; M, male; m, meter; mg, milligram; min, minute; ml, milliter; SLE, systemic lupus erythematosus.

In terms of immunosuppressive therapy, the induction agent used the most in the entire cohort was cyclophosphamide (40.1% of the patients), with doses and intervals of administration either as in the NIH protocol or Eurolupus protocol at the discretion of the treating physician, followed by mycophenolate mofetil (18.3% of the patients). In terms of the maintenance therapy, mycophenolate mofetil (43.88% of the patients) was most frequently used, followed by azathioprine (12.95% of the patients), but also combinations of mycophenolate mofetil or azathioprine and calcineurin inhibitors or rituximab (17.26% of the patients) (**Table 2**).

**Table 2:** Histological and treatment characteristics of the study cohort.

Variable	Value	Variable	Value
*Class of LN (entire cohort)*		*Induction regimen*	
I	4.76%	Cyclophosphamide	40.1%
II	10.88%	MMF	18.3%
III ± V	25.86%	Azathioprine	4.2%
IV ± V	42.18%	MMF/Azathioprine + CNI	2.1%
V	12.24%	Rituximab	3.5%
VI	2.04%	Steroids monotherapy	7.04%
TMA	1.36	MMF/Cyclophosphamide + Rituximab	16.2%
Lupus podocytopathy	0.68%	Belimumab/Methotrexate	2.81%
*NIH indices (min-max)*		*Maintenance regimen*	
Activity Index	5 (0-20)	MMF	43.88%
Chronicity Index	3 (0-10)	Azathioprine	12.95%
*Class of LN (biopsy prot. < 0.5 g/d)*		MMF/Azathioprine + CNI	7.19%
I	11.8%	Rituximab	1.44%
II	35.3%	Steroids monotherapy	12.23%
III ± V	11.8%	MMF/Azathioprine + Rituximab	10.07%
IV ± V	23.4%	Methotrexate	0.72%
V	11.8%	Anifrolumab	12.3%
VI	0%		
TMA	5.9%		
*NIH indices (min-max)*			
Activity Index	1 (0-14)		
Chronicity Index	0 (0-6)		

CNI, calcineurin inhibitors; d, day; LN, lupus nephritis; max, maximum; min, minimum; MMF, mycophenolate mofetil; NIH, National Institutes of Health; prot., proteinuria.

The treatment strategy has changed over the years as represented by the distribution of induction treatment used before and after 2020. After 2020, the proportion of patients that has received an Euro-Lupus induction protocol has increased; also, the administration of mycophenolate mofetil as an induction agent has increased. Moreover, there has been a rise in the choice of the treating physicians of combination therapies as induction treatment, consistent with the emergence of evidence from more recent clinical trials. Similarly, during maintenance therapy, there has been a rise in the use of mycophenolate mofetil and the combination therapies based on mycophenolate mofetil or azathioprine and calcineurin inhibitors or rituximab. Also, after 2020 the administration of rituximab (p 0.019) and corticosteroids (p 0.008) has increased either during induction or maintenance. The treatment regimens used are summarized in **Figure 1**.

**Figure 1:**
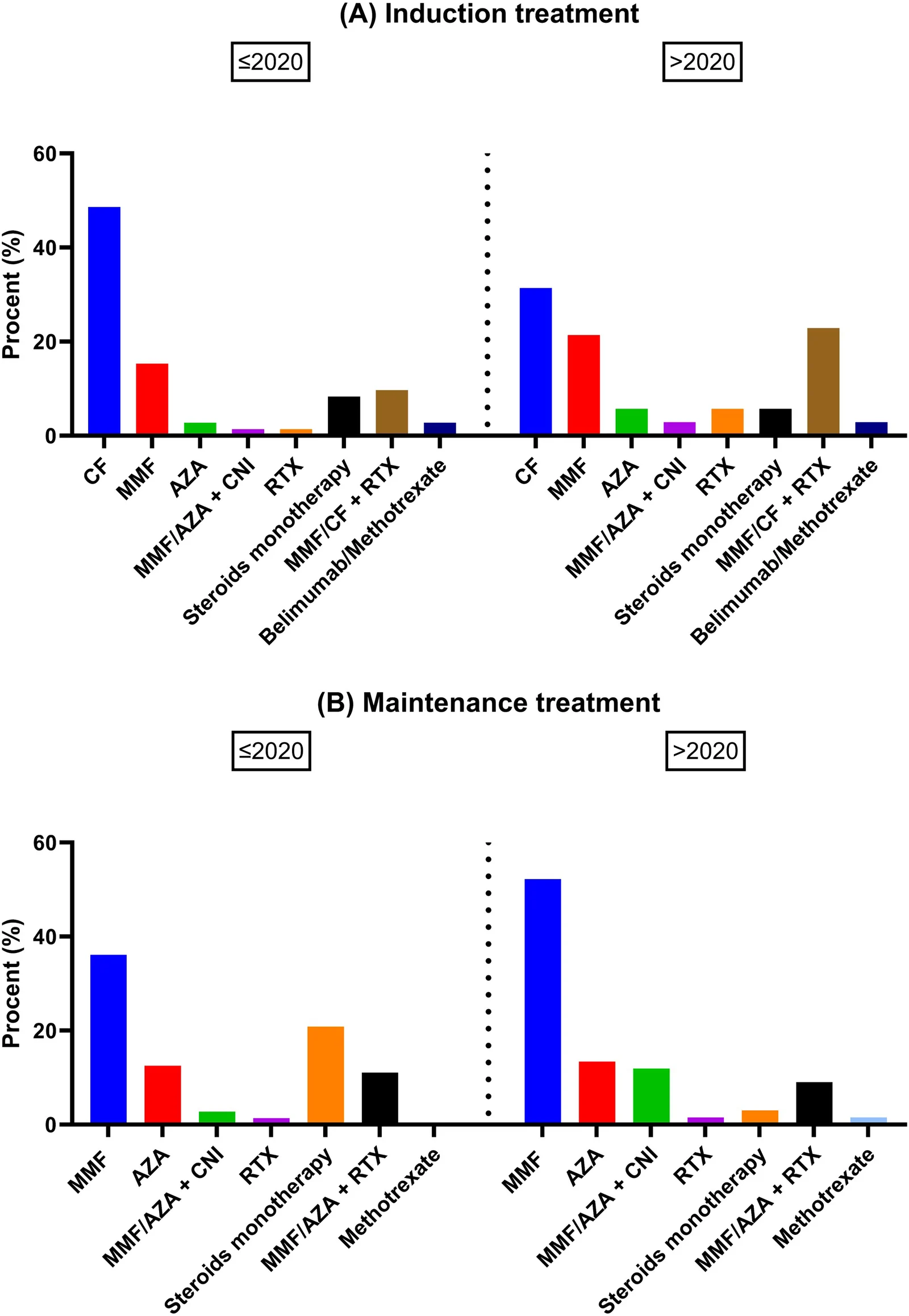
Distribution of treatment regimens. **(A)** Induction treatment (≤2020 and >2020). **(B)** Maintenance treatment (≤2020 and >2020). AZA, azathioprine; CF, cyclophosphamide; CNI, calcineurin inhibitors; MMF, mycophenolate mofetil; RTX, rituximab.

In terms of histological features, 68.04% of the patients had proliferative class III or IV LN, in association or not with class V LN. 12.24% presented isolated class V LN, and 15.64% were having a class I or II LN. 2 patients had isolated thrombotic microangiopathy, and 1 patient had isolated podocytopathy. A proportion of 2.04% of the patients had a class VI LN at baseline. The median activity index (AI) and chronicity index (CI) were 5 (minimum (min)-maximum (max): 0-20), respectively 3 (min-max: 0-10) (**Table 2**).

From the entire cohort, 17 patients had a renal biopsy done at a proteinuria level of less than 500 mg/day. 35.2% of these patients had a proliferative LN with or without an associated class V LN. The median AI was 1, but we observed that the maximum AI identified was 14 in this subgroup, which suggests that we can identify severe cases of proliferative LN even in patients with proteinuria ≤ 0.5 g/day. Another important observation is that no patient has progressed or died during the follow-up period of 62 months in this subgroup.

The entire cohort had a median follow-up period of 39.5 months (IQR: 16.7-72). The entire cohort had a kidney/general survival of 91% at 1 year, 88% at 2 years, 82% at 5 years, and 69% at 10 years (**Figure 2A**); 10.3% of them presented a doubling of serum creatinine, 11% developed ESKD, and 6.8% died during the follow-up period. 17.1% reached the combined event during the follow-up period (**Supplementary Figure S1**). Patients with proliferative LN with or without associated class V LN presented a worse prognosis, with 10.1% reaching the end-point of doubling of serum creatinine, 14.1% arriving at ESKD, and 7.1% dying during the follow-up period. Also, the combined endpoint was reached in 19.2% of the cases. In the subgroup of patients with proteinuria more than 3 g/day, 19.6% of them presented doubling of serum creatinine, 21.6% arrived at ESKD, 11.8% died, and 31.4% reached the combined event during follow-up; this subgroup of patients had the worst prognosis.

**Figure 2:**
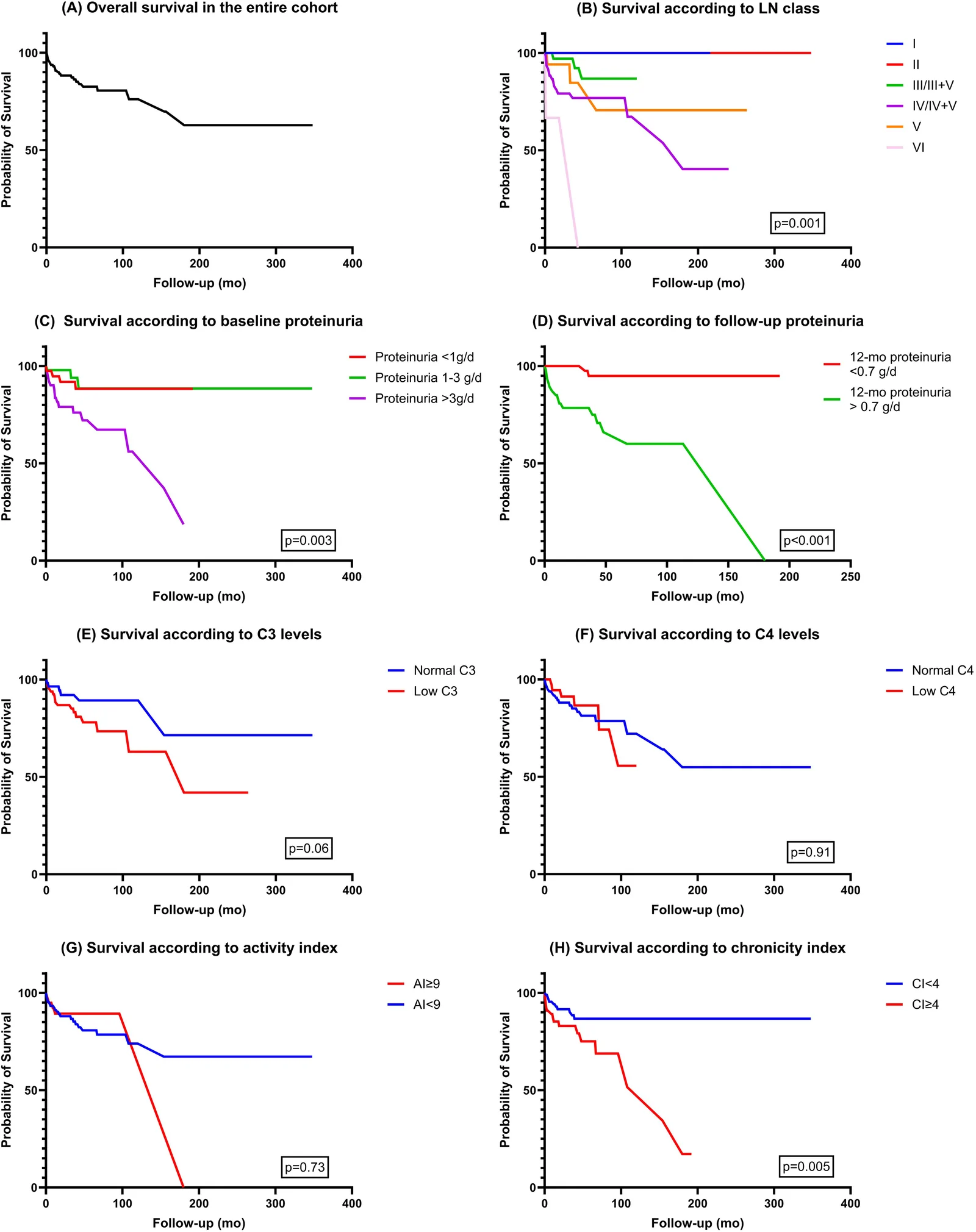
Overall survival (renal and patient survival) in the entire cohort and stratified according to different clinical or histological variables. **(A)** Overall survival in the entire cohort. **(B)** Survival according to LN class. **(C)** Survival according to baseline proteinuria. **(D)** Survival according to follow-up proteinuria. **(E)** Survival according to C3 levels. **(F)** Survival according to C4 levels. **(G)** Survival according to activity index H. Survival according to chronicity index. LN, lupus nephritis.

From the entire cohort, 103 patients had an available proteinuria evaluation at 12 months. In the entire cohort, 49.1% and 54.4% of the patients achieved a proteinuria of <0.7 g/24-h at 6 and 12 months, respectively. Among patients with proliferative classes of LN, 44.4% achieved a proteinuria level of <0.7g/day at 6 months, increasing to 48% at 12 months of follow-up. 40% and 45.9% of the patients with proteinuria >1g/day at baseline reached a proteinuria of <0.7g/day at 6 and 12 months, respectively. Patients with baseline proteinuria > 3g/day needed longer periods of follow-up to respond, with only 16.7% and 25% achieving proteinuria <0.7 g/day at 6 and 12 months, respectively (**Supplementary Figure S2**).

### 3.2 Predictive factors for combined event- Kaplan-Meier analysis

Kaplan-Meier analysis showed that patients with class V LN, proliferative LN with or without class V LN, and class VI LN had significantly worse survival compared with class I or II (p= 0.001) (**Figure 2B**).

Patients with baseline proteinuria more than 3 g/day had significantly lower survival compared with those with proteinuria 1–3 g/day (p= 0.003). Also, the subgroup of patients that still had a proteinuria >0.7g/day at 12 months had worse survival than those with proteinuria <0.7g/day at 12 months (p<0.001) (**Figures 2C, D**).

The subgroup of patients with low C3 at baseline tended to have a worse prognosis compared to those with normal C3 (p=0.06). The difference between normal and low C4 at baseline did not reach statistical significance (p=0.91) (**Figures 2E, F**).

For the analysis based on the AI, we have divided the patients in 2 groups: with AI ≥9 and with AI <9. The subgroup of patients with a higher histologic activity had a better prognosis when we look at the initial period of the Kaplan-Meier curve (p=0.73); in the entire period of follow-up, there is a patient who had a longer period of follow-up and has progressed. For the analysis based on CI, we have divided the patients into 2 groups: with CI ≥4 and with CI <4. The subgroup of patients with a higher CI had a worse prognosis during the period of follow-up (p= 0.005) (**Figures 2G, H**).

### 3.3 Predictive factors for combined event- univariate and multivariate analysis of the entire cohort

The results of the univariate Cox proportional hazards regression analysis of the entire cohort are presented in **Supplementary Tables 1** and **2**.

We first evaluated the entire cohort using a baseline model including only variables collected at the moment of biopsy (**Supplementary Table 2**). In this multivariate Cox proportional hazards regression analysis, we identified the following independent risk factors for the combined event: age>50 years old (HR 3.75, 95% CI 1.17-11.9, p=0.02), proteinuria ≥ 3 g/day (HR 3.42, 95% CI 1.33-8.74, p=0.01) and Hb of <10 g/dl (HR 5.97, 95% CI 2.3-15.4, p<0.001). In contrast, we identified the following tendencies for protective factors for the combined event in the entire cohort: AI ≥9 (HR 0.33, 95% CI 0.1-1.1, p=0.07) and low C4 (HR 0.29, 95% CI 0.07-1.1, p=0.07) (**Figure 3A**).

**Figure 3:**
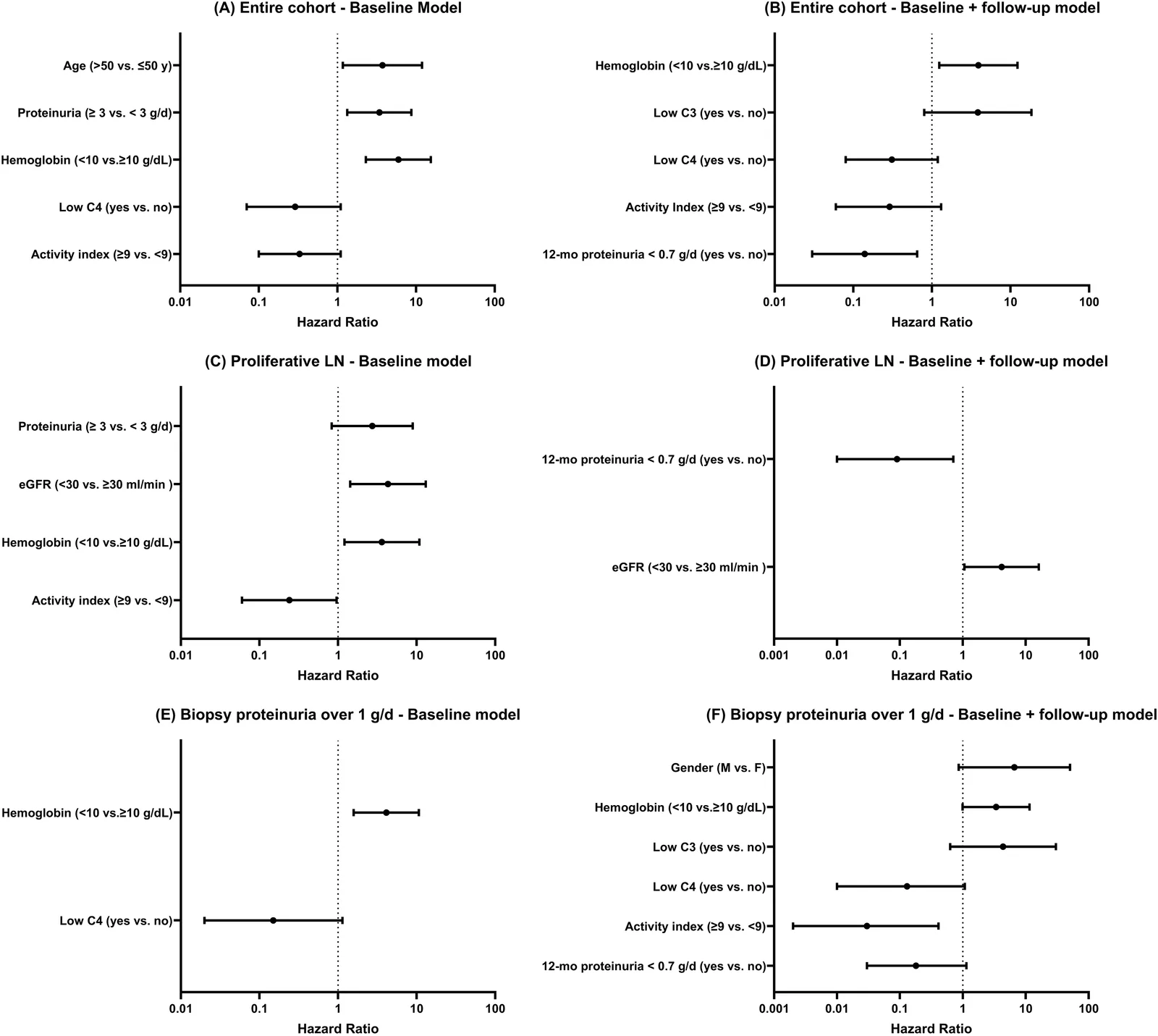
Multivariate Cox regression analysis regarding factors predictive of the composite endpoint. **(A)** Entire cohort- Baseline Model. **(B)** Entire cohort- Baseline + follow-up model. **(C)** Proliferative LN- Baseline model. **(D)** Proliferative LN- Baseline + follow-up model. **(E)** Biopsy proteinuria over 1g/d – Baseline Model. **(F)** Biopsy proteinuria over 1g/d- Baseline+ follow-up model. eGFR, estimated glomerular filtration rate; F, female; LN, lupus nephritis; y, years; 12-m, 12-months; M, male.

When proteinuria <0.7 g/day at 12 months was incorporated into the baseline model, the significance of proteinuria at baseline was lost. In this model, the only significant negative predictor that remained was Hb <10 g/dl (HR 3.91, 95% CI 1.24-12.3, p=0.02). Low C3 showed a trend as a negative predictor in this model (HR 3.85, 95% CI 0.8-18.5, p=0.09). Proteinuria <0.7g/day at 12 months was a strong protective factor for the combined event (HR 0.14, 95% CI 0.03-0.65, p=0.01). We identified a trend toward significance for low C4 as a protective factor (HR 0.31, 95% CI 0.08-1.19, p=0.09). (**Figure 3B**).

### 3.4 Predictive factors for combined event- univariate and multivariate analysis of the subgroup of patients with proliferative LN

Cox proportional hazards regression univariate analysis of the subgroup of patients with proliferative LN is represented in **Supplementary Table 2**.

We also performed a multivariate Cox proportional hazards regression analysis in the subgroup of patients with proliferative LN, initially including only baseline factors (**Supplementary Table 2**). In the baseline model, the following negative predictors for the combined event were identified: eGFR <30 ml/min/1.73 m^2^ (HR 4.33, 95% CI 1.43-13.1, p=0.009) and Hb <10 g/dl (HR 3.62, 95% CI 1.21-10.87, p=0.02). Also, there was a trend toward significance for proteinuria ≥3g/day as a negative predictor (HR 2.73, 95% CI 0.83-8.94, p=0.09). In this subgroup, we identified a protective factor to be an AI ≥9 (HR 0.24, 95% CI 0.06-0.96, p=0.04) (**Figure 3C**). When proteinuria <0.7 g/day at 12 months was added to the baseline model, the baseline proteinuria lost its predictive tendency, and the only significant negative predictor was eGFR <30 ml/min/1.73 m^2^ (HR 4.15, 95% CI 1.06-16.2, p=0.04). Proteinuria <0.7 g/day at 12 months was a strong protective factor in this subgroup (HR 0.09, 95% CI 0.01-0.71, p=0.02) (**Figure 3D**).

### 3.5 Predictive factors for combined event- univariate and multivariate analysis of the subgroup of patients with proteinuria at baseline >1g/day

Cox proportional hazards regression univariate analysis of the subgroup of patients with proteinuria at baseline >1g/day is represented in **Supplementary Table 2**.

In the multivariate baseline model, the only significant negative predictor was Hb <10g/dl (HR 4.12, 95% CI 1.58-10.7, p=0.004), while low C4 showed a trend toward protection (HR 0.15, 95% CI 0.02-1.14, p=0.06) (**Figure 3E**). When proteinuria at 12 months was incorporated into the baseline model, we observed that an Hb of <10g/dl at baseline remained a negative predictor (HR 3.39, 95% CI 0.99-11.5, p=0.05), and male sex showed a trend toward being a negative predictor (HR 6.6, 95% CI 0.86-50.7, p=0.07). Also, we found that an AI of ≥9 (HR 0.03, 95% CI 0.002-0.41, p 0.009) and proteinuria <0.7g/day at 12 months (HR 0.18, 95%CI 0.03-1.14, p=0.07) were associated with a reduced risk of the composite outcome, the latter showing only a trend toward significance (**Figure 3F**).

### 3.6 Prognostic score

We have selected the predictors identified previously for the composite outcome and given a score of 1 for each negative predictor and -1 for each protective factor (**Supplementary Table S3**). Patients were stratified into 3 risk categories according to the total score: low risk (total score ≤0), intermediate risk (total score 1-2), and high risk (total score >2). When we have included only the factors at baseline, this prognostic score proved an AUC of 0.82 (p<0.0001). The AUC rose to 0.86 (p<0.001) and the difference between the 3 categories became statistically significant (no matter the comparison: high-low risk; intermediate-low risk; or high-intermediate risk) when we included in this model also the proteinuria at 12 months (**Supplementary Figure S3**).

## 4 Discussions

In this retrospective cohort study of biopsy-proven LN, we evaluated long-term renal and patient outcomes, identified predictors of adverse prognosis, and developed a prognostic score integrating clinical, laboratory, and histologic parameters.

The definition of the primary end-point used in the BLISS-LN trial included the achievement of a urine protein-creatinine ratio of 0.7 g/g or less, which proved in earlier studies to be an independent predictor of long-term outcome of the renal function (4, 26, 27). In this cohort of biopsy-proven LN, 49.1% and 54.4% of the patients achieved a proteinuria <0.7 g/day at 6 months, respectively, at 12 months. Early achieving and maintaining renal response proved to be protective in the study conducted by Michelakis, where each additional year on sustained CRR reduced the risk of the severe decline of renal function by 19% and the risk of composite adverse outcome of ESKD or death by 25% (7). Also, in a recently published cohort of Chinese LN patients, achieving modified PERR and modified CRR at 2 years post-biopsy predicted risk reductions in kidney adverse outcomes (28).

The overall survival rate of the entire cohort was 91% at 1 year, 88% at 2 years, 82% at 5 years, and 69% at 10 years. 11% of our cohort reached ESKD, and 6.8% of them died, with 17.1% of the entire cohort reaching the combined event during the follow-up period. In other cohorts published in the literature in the last decade, the rate of reaching ESKD was 2.9%-20.2% (16, 29–35) and for death of 5-20.2% (10, 16, 28, 31, 32, 34, 36).

As expected, a higher CI was identified as a predictor for advanced renal dysfunction/ESKD in multiple cohorts of LN patients (3, 14, 16, 17, 29, 31, 37), as well as the presence of interstitial fibrosis/tubular atrophy (30, 33, 38, 39). Proteinuria at baseline was found to be an independent predictor of adverse kidney outcomes, as suggested also by our group in 2018, along with both active and chronic histological parameters (the presence of crescents, global glomerulosclerosis, tubulitis, and adhesions) (15). In the multicentric study of 499 LN patients conducted by Moroni, high AI proved to be an independent predictor of ESKD (3); also, an AI>20 was associated with a higher risk of incident ESKD in a cohort of 379 patients (14). Conversely, in other cohorts, AI was not associated with an increased ESKD risk for LN patients (16). In the study conducted by Ryjnink, a progressive eGFR decline and ESKD were independently predicted by fibrinoid necrosis; in contrast, wire loops and endocapillary lymphocytes predicted eGFR recovery during a 10-year follow-up (39). The limited prognostic value of AI might be explained by the heterogeneity of the active lesions, the exclusion of lupus podocytopathy and vascular lesions as thrombotic microangiopathy or lupus vasculitis, and the lower scores assigned to the tubulointerstitial active lesions (40). In our study, those patients who had a high AI had a better renal prognosis, although not statistically significant, because there was a patient who had a long follow-up and had progressed. This seemingly paradoxical finding likely reflects the differential biological significance of active versus chronic lesions. Active inflammatory lesions are potentially reversible and may respond to aggressive induction therapy, whereas chronic lesions represent permanent damage and portend progressive renal dysfunction. Our findings align with studies suggesting that chronicity, rather than activity, drives long-term renal decline, and underscore the importance of integrating histologic chronicity into prognostic assessment and therapeutic decision-making.

Low C3 was found to be predictive of adverse kidney outcomes/death in 2 cohorts in the last decade (41, 42); C4 hypocomplementemia was shown to correlate with histologic activity and be a potential disease activity and renal flares predictor in SLE cohorts (13), but no cohort reported the potential protective value of baseline low C4 in LN patients for their long-term renal outcome. This observation may reflect differential activation of complement pathways in LN. While immune complex–mediated injury is initiated through the classical pathway, sustained tissue damage may be driven by alternative pathway overactivation, as suggested by the association between low C3 and renal flares (43). The potential protective association of low C4 warrants further investigation but may reflect heightened immunologic activity responsive to immunosuppression rather than irreversible damage.

Achieving proteinuria <0.7g/day at 12 months emerged as a strong protective factor for the combined event both in the entire cohort and in the subgroup of proliferative LN patients, as seen also in other LN cohorts (26, 27, 44). In our cohort, after inclusion of proteinuria < 0.7g/day in the multivariate analysis baseline model, baseline proteinuria lost statistical significance, which suggests that the kinetics of proteinuria is more important than its baseline value, and the clinical response is more important than the initial clinical syndrome. 76 patients from the Euro-Lupus Nephritis trial cohort had an available serum creatinine measurement 7 years after their entry into the trial; in this cohort, achieving an early proteinuria reduction to <0.8 g/day 12 months post-randomization proved to be the best short-term predictor of good long-term renal outcome, showing an AUC of 0.83, a sensitivity of 81%, a specificity of 78%, a positive predictive value of 88% and a negative predictive value of 67% (27). Also, the analysis of 90 patients included in the MAINTAIN Nephritis Trial proved that a target absolute proteinuria value <0.7g/day after 12 months of treatment is the best predictor for a serum creatinine ≤ 1.0 mg/dl after at least 7 years of follow-up in LN patients, with a sensitivity of 71%, a specificity of 75%, a positive predictive value of 94%, but a low negative predictive value of 31% (26). In both trials, adding hematuria ≤5 red blood cells/hpf at 12 months reduced the sensitivity of a proteinuria <0.7-0.8 g/day at 12 months after randomization to 41%-47% (26, 27). Based on these observations, the cut-off value of urinary protein to creatinine of 0.7-0.8 g/day or less was included in the definitions of renal end-points in the latest LN trials (4, 6).

A recent publication proposes a clinical prediction model named Score Mestizo based on only two baseline parameters: eGFR <30 ml/min/m^2^ and CI>2, with the aim to better stratify the patients in risk categories of adverse renal outcome and accordingly to better adjust immunosuppression and interval of monitoring of these patients (17). Based on their analysis, patients included in the high risk group present a HR of achieving the adverse outcome earlier of 3.22 (95% CI 1.6406.32, p 0.001) when compared with the patients from the intermediate risk group, and a HR of 7.53 (95% CI 3.89-14.76, p <0.001) when compared with those from the low risk group; this model has been externally validated in a Mexican cohort (17). Also, based on our predictors identified for the composite outcome, we aimed to develop a prognostic model to estimate the risk of poor renal and general outcomes in LN patients. Our baseline prognostic model showed an AUC of 0.82; when we also included proteinuria at 12 months, the AUC rose to 0.86. This prognostic score has to be validated in independent cohorts.

The strengths of this study include a well-characterized, biopsy-proven LN cohort with long-term follow-up and comprehensive clinical and histologic data. Limitations include the retrospective, single-center design, important cofounding factors not included as covariates (e.g., arterial hypertension or diabetes mellitus), potential impact of treatment changes during follow-up, potential treatment heterogeneity over the long study period, and the lack of external validation of the prognostic score. Additionally, evolving therapeutic strategies may limit the generalizability of findings to current treatment paradigm. Furthermore, competing risks, such as mortality from other causes, were not formally evaluated in the survival analysis. Second, potential time-varying exposures, including subsequent treatment changes or intercurrent events during follow-up, were not incorporated as time-dependent covariates due to sample size constraints and treatment heterogeneity. Future larger studies are needed to address these dynamics.

## 5 Conclusions

In our biopsy-proven LN cohort, 11% arrived to ESKD, and 6.8% died during follow-up. We found that age >50 years old, proteinuria ≥ 3g/day, hemoglobin <10 g/dl, and low baseline C3 (tendency) are predictive for the combined event composed of doubling of serum creatinine, ESKD, or death in the entire cohort. Having a low baseline C4 (tendency), an AI ≥ 9 (tendency), and achieving proteinuria <0.7g/day at 12 months emerged as protective factors for the combined event in the entire cohort. Our findings support a shift from baseline-centered risk assessment toward dynamic response-based prognostication in lupus nephritis and provide the basis for a clinically applicable prognostic score that requires validation in independent, external cohorts.

## Data Availability

The raw data supporting the conclusions of this article will be made available by the authors, without undue reservation.
