# Supplementary material for: Predictors of renal and patient survival in lupus nephritis: a 28-year single-center cohort study
Source: Front Nephrol. 2026 Sep 25;6:1945616. doi: 10.3389/fneph.2026.1945616 (PMC13649432; doi:10.3389/fneph.2026.1945616)
Supplement: Supplementary file 1 [file Table1.docx]

Supplementary Material

**Supplemental Table 1. Univariate analysis regarding factors associated with the composite endpoint.**

| **Variable** | **With composite outcome** | **Without composite outcome** | **p-value** |
| --- | --- | --- | --- |
| **Number of patients** | 25 | 122 |  |
| **Age (years)** | 37 (IQR:24.5-50) | 32 (IQR:24-41) | 0.18 |
| **Age over 50 years (%)** | 24% | 16.5% | 0.39 |
| **Gender (%F/%M)** | 72%/28% | 81.8%/18.2% | 0.27 |
| **Time since SLE onset (years)** | 1.62 (IQR:0-9.75) | 2 (IQR:0.5-8) | 0.53 |
| **Antiphospholipid syndrome (%)** | 20% | 25.4% | 0.56 |
| **Serum creatinine (mg/dL)** | 1.5 (IQR:1.17-2.99) | 0.93 (IQR: 0.74-1.21) | <0.001 |
| **eGFR (mL/min/1.73 m^2^)** | 49 (IQR: 20.5-63.5) | 86 (IQR:60.6-114) | <0.001 |
| - **eGFR < 30 mL/min (%)** | 3.3% | 36% | <0.001 |
| - **eGFR < 60 mL/min (%)** | 68% | 24% | <0.001 |
| **Serum albumin (g/dL)** | 3.3 (IQR:2.5-3.7) | 3.9 (IQR:3.3-4.2) | <0.001 |
| **Serum albumin < 3 g/dL (%)** | 36% | 13.3% | 0.01 |
| **Hemoglobin (g/dL)** | 9.7 (IQR:8.6-11.8) | 12.1 (IQR:10.3-13) | <0.001 |
| **Hemoglobin < 10 g/dL (%)** | 60% | 21.5% | <0.001 |
| **24-h proteinuria (g/day)** | 4.5 (IQR:1.82-7) | 1.69 (IQR:0.8-4.2) | 0.002 |
| **24-h proteinuria (%)** |  |  |  |
| - **< 1 g/day** | 17.4% | 30.4% | 0.002 |
| - **1-3 g/day** | 13% | 39.1% |  |
| - **>3 g/day** | 69.6% | 30.4% |  |
| **Microscopic hematuria (%)** | 68% | 66.1% | 0.85 |
| **Serum C3 (mg/dL)** | 78 (IQR:64-98) | 84.5 (IQR:62.9-106.7) | 0.19 |
| **Low serum C3 (%)** | 73.9% | 56% | 0.11 |
| **Serum C4 (mg/dL)** | 19.4 (IQR:11-27.8) | 15 (IQR:7.7-20.8) | 0.1 |
| **Low serum C4 (%)** | 17.4% | 30.8% | 0.19 |
| **Activity Index** | 5 (IQR:3-8) | 4 (IQR:1-9) | 0.39 |
| **High Activity Index** | 20% | 28.9% | 0.36 |
| **Chronicity Index** | 4 (IQR:1-6.5) | 2 (IQR:0-4) | 0.01 |
| **High Chronicity Index** | 60% | 33.1% | 0.01 |

eGFR: estimated glomerular filtration rate; F: female; IQR: interquartile range; M: male; SLE: systemic lupus erythematosus.
