# Supplementary material for: Predictors of renal and patient survival in lupus nephritis: a 28-year single-center cohort study
Source: Front Nephrol. 2026 Sep 25;6:1945616. doi: 10.3389/fneph.2026.1945616 (PMC13649432; doi:10.3389/fneph.2026.1945616)
Supplement: Supplementary file 2 [file Table2.docx]

Supplementary Material

**Supplemental Table 2. Cox regression analysis regarding factors predictive of the composite endpoint.**

| **Variable** | **Univariate analysis** | | **Multivariate analysis***  **Baseline Model** | | **Multivariate analysis***  **Baseline + Follow-up Model** | |
| --- | --- | --- | --- | --- | --- | --- |
|  | **Hazard ratio**  **(95%CI)** | **p-value** | **Hazard ratio**  **(95%CI)** | **p-value** | **Hazard ratio**  **(95%CI)** | **p-value** |
| **(A) Entire cohort** | | | | | | |
| Gender (male vs. female) | 1.71 (0.71-4.09) | 0.23 | - | - | - | - |
| Age (>50 vs. ≤50 y) | 2.41 (0.93-6.23) | 0.07 | 3.75 (1.17-11.9) | 0.02 | - | - |
| Proteinuria (≥ 3 vs. < 3 g/d) | 4.11 (1.68-9.99) | 0.002 | 3.42 (1.33-8.74) | 0.01 | - | - |
| eGFR (<30 vs. ≥30 ml/min) | 5.99 (2.57-13.96) | <0.001 | - | - | - | - |
| Hemoglobin (<10 vs.≥10 g/dL) | 4.14 (1.85-9.26) | 0.001 | 5.97 (2.3-15.4) | <0.001 | 3.91 (1.24-12.3) | 0.02 |
| Hematuria (yes vs. no) | 1.04 (0.45-2.43) | 0.91 | - | - | - | - |
| Low C3 (yes vs. no) | 2.36 (0.93-6.02) | 0.07 | - | - | 3.85 (0.8-18.5) | 0.09 |
| Low C4 (yes vs. no) | 0.61 (0.21-1.83) | 0.38 | 0.29 (0.07-1.1) | 0.07 | 0.31 (0.08-1.19) | 0.09 |
| APS (yes vs. no) | 0.86 (0.32-2.31) | 0.77 | - | - | - | - |
| Activity index (≥9 vs. <9) | 0.84 (0.31-2.26) | 0.73 | 0.33 (0.1-1.1) | 0.07 | 0.29 (0.06-1.31) | 0.11 |
| Chronicity index (≥4 vs. <4) | 2.96 (1.32-6.64) | 0.008 | - | - | - | - |
| 12-mo prot. <0.7 g/d (yes vs. no) | 0.09 (0.02-0.42) | 0.002 | - | - | 0.14 (0.03-0.65) | 0.01 |
| Time since SLE onset (years) | 1.00 (0.94-1.05) | 0.98 | - | - | - | - |
| Year of biopsy (>2020 vs. ≤2020) | 0.55 (0.21-1.45) | 0.23 | - | - | - | - |
| **(B) Proliferative lupus nephritis** | | | | | | |
| Gender (male vs. female) | 1.19 (0.42-3.33) | 0.74 | - | - | - | - |
| Age (>50 vs. ≤50 y) | 2.1 (0.67-6.58) | 0.2 | - | - | - | - |
| Proteinuria (≥ 3 vs. < 3 g/d) | 3.15 (1.01-9.76) | 0.04 | 2.73 (0.83-8.94) | 0.09 | - | - |
| eGFR (<30 vs. ≥30 ml/min) | 5.5 (2.12-14.24) | <0.001 | 4.33 (1.43-13.1) | 0.009 | 4.15 (1.06-16.1) | 0.04 |
| Hemoglobin (<10 vs.≥10 g/dL) | 3.79 (1.48-9.67) | 0.005 | 3.62 (1.21-10.87) | 0.02 | - | - |
| Hematuria (yes vs. no) | 1.01 (0.36-2.82) | 0.97 | - | - | - | - |
| Low C3 (yes vs. no) | 3.95 (1.07-14.5) | 0.04 | - | - | - | - |
| Low C4 (yes vs. no) | 0.66 (0.21-2.09) | 0.48 | - | - | - | - |
| APS (yes vs. no) | 0.68 (0.19-2.35) | 0.54 | - | - | - | - |
| Activity index (≥9 vs. <9) | 0.64 (0.23-1.8) | 0.4 | 0.24 (0.06-0.96) | 0.04 | - | - |
| Chronicity index (≥4 vs. <4) | 1.38 (0.56-3.41) | 0.48 | - | - | - | - |
| 12-mo prot. <0.7 g/d (yes vs. no) | 0.08 (0.1-0.64) | 0.01 | - | - | 0.09 (0.1-0.71) | 0.02 |
| **(C) Biopsy proteinuria over 1 g/d** | | | | | | |
| Gender (male vs. female) | 1.55 (0.58-4.09) | 0.37 | - | - | 6.6 (0.86-50.7) | 0.07 |
| Age (>50 vs. ≤50 y) | 0.92 (0.21-4.08) | 0.91 | - | - | - | - |
| eGFR (<30 vs. ≥30 ml/min) | 3.9 (1.47-4.08) | 0.006 | - | - | - | - |
| Hemoglobin (<10 vs.≥10 g/dL) | 4.31 (1.68-11) | 0.002 | 4.12 (1.58-10.7) | 0.004 | 3.39 (0.99-11.5) | 0.05 |
| Hematuria (yes vs. no) | 0.84 (0.3-2.35) | 0.74 | - | - | - | - |
| Low C3 (yes vs. no) | 2.54 (0.83-7.76) | 0.1 | - | - | 4.37 (0.63-30.2) | 0.13 |
| Low C4 (yes vs. no) | 0.15 (0.02-1.17) | 0.07 | 0.15 (0.02-1.14) | 0.06 | 0.13 (0.01-1.08) | 0.06 |
| APS (yes vs. no) | 0.67 (0.19-2.31) | 0.52 | - | - | - | - |
| Activity index (≥9 vs. <9) | 0.42 (0.12-1.46) | 0.17 | - | - | 0.03 (0.002-0.41) | 0.009 |
| Chronicity index (≥4 vs. <4) | 1.99 (0.79-5) | 0.14 | - | - | - | - |
| 12-mo prot. <0.7 g/d (yes vs. no) | 0.2 (0.04-0.91) | 0.03 | - | - | 0.18 (0.02-1.14) | 0.07 |

*Abbreviations: APS: antiphospholipid syndrome; CI: confidence interval; eGFR: estimated glomerular filtration rate; mo: months; prot.: proteinuria; SLE: systemic lupus erythematosus; y: year;*

*** **Backward stepwise model;* All variables from the univariate analysis were entered into the initial model. At each step, variables were progressively removed from the model based on the highest p-value
