# Supplementary material for: Predictors of renal and patient survival in lupus nephritis: a 28-year single-center cohort study
Source: Front Nephrol. 2026 Sep 25;6:1945616. doi: 10.3389/fneph.2026.1945616 (PMC13649432; doi:10.3389/fneph.2026.1945616)
Supplement: Supplementary file 3 [file Table3.docx]

Supplementary Material

**Supplemental Table 3. Risk-stratifying algorithm.**

| **Variable** | **Score** |
| --- | --- |
| **eGFR < 30 ml/min** | 1 |
| **Proteinuria > 3 g/d** | 1 |
| **Low C3** | 1 |
| **Low C4** | -1 |
| **Hemoglobin < 10 g/dL** | 1 |
| **Activity index ≥9** | -1 |
| **Chronicity index ≥4** | 1 |
| **12-mo proteinuria < 0.7 g/d*** | -1 |
|  |  |
| **Baseline Score** |  |
| - **Low risk** | ≤0 |
| - **Intermediate risk** | 1-2 |
| - **High risk** | >2 |
|  |  |
| **Baseline + Follow-up Score** |  |
| - **Low risk** | ≤0 |
| - **Intermediate risk** | 1-2 |
| - **High risk** | >2 |

*Incorporated in the baseline + follow-up score

*Abbreviations: d: day; dL: deciliter; eGFR: estimated glomerular filtration rate; g: gram; mo: months*
